# Pathology, bacteriology and molecular studies on caseous lymphadenitis in *Camelus dromedarius* in the Emirate of Abu Dhabi, UAE, 2015-2020

**DOI:** 10.1371/journal.pone.0252893

**Published:** 2021-06-08

**Authors:** Abdelnasir Mohammed Adam Terab, Ghada El Derdiri Abdel Wahab, Hassan Zackaria Ali Ishag, Nasereldien Altaib Hussein Khalil, El Tigani Ahmed El Tigani-Asil, Farouk Mohamed Hashem, Abdelmalik Ibrahim Khalafalla, Asma Abdi Mohamed Shah, Salama Suhail Mohammed Al Muhairi

**Affiliations:** Veterinary Laboratories, Animal Wealth Sector, Abu Dhabi Agriculture and Food Safety Authority (ADAFSA), Abu Dhabi, United Arab Emirates (UAE); University of Lincoln, UNITED KINGDOM

## Abstract

Caseous lymphadenitis (CLA) or pseudotuberculosis is a chronic zoonotic bacterial disease caused by *Corynebacterium pseudotuberculosis*, which affects livestock and humans. This study aimed to describe the pathology, bacteriology and confirm the identity of the pathogen by 16S rRNA gene sequencing in Camelus dromedarius. A total of 12 camels with suspected CLA in three regions of Abu Dhabi Emirate (Abu Dhabi, Al Ain and Al Dhafra), United Arab Emirate (UAE) were subjected to clinical and postmortem examinations from January 2015 to December 2020. Clinically, camels were emaciated and showed the presence of external caseous abscesses suggestive of CLA. Postmortem examination showed multiple abscesses of variable sizes with caseous material encapsulated by fibrous tissue in the liver, lungs, muscle, and lymph nodes. Following clinical and postmortem examination, blood, pus and different tissue samples were collected for subsequent analysis. Histopathological examination of all organs stained with Hematoxylin and Eosin (H&E) indicated a central caseo-necrotic core that was admixed with bacterial colonies and infiltration of chronic inflammatory cells, surrounded by a pyogenic membrane, and an outer fibrous connective tissue capsule. Bacterial culture identified the isolates of *Corynebacterium pseudotuberculosis* biotype ovis strain, and these isolates were shown to be sensitive to all antibiotics tested (penicillin, ampicillin, Co-trimoxazole, enrofloxacin and tetracycline). Moreover, the identity of the isolates was confirmed by partial sequencing of the 16S rRNA gene which showed a 100% identity to *Corynebacterium pseudotuberculosis*. Phylogenetic analysis based on 16S rRNA gene sequence clearly differentiates *Corynebacterium pseudotuberculosis* from other species of *Corynebacterium*. Briefly, this study provided the basic information for infection of *Corynebacterium pseudotuberculosis* in Camels and will help in controlling of this pathogen in the region.

## Introduction

Caseous lymphadenitis (CLA) is a chronic, contagious and non-fatal bacterial infection of livestock. It mostly affects sheep and goats, cattle, horses, buffalo, camelids, deer [1–5], and humans [6,7], thus, it is considered a zoonotic disease.

The disease is caused mainly by a Gram positive intracellular coccobacilli bacteria [8,9] called *Corynebacterium pseudotuberculosis* previously known as *Corynebacterium ovis*. Currently, there are two biovars of *Corynebacterium pseudotuberculosis* based on the production of nitrate reductase; the first one is a nitrate negative (biovar ovis) which infects mainly sheep and goats and the second one is a nitrate positive (biovar equi) which infects horses and cattle [1,4]. Camels are reported to be infected by both biovars especially in the co-herd breeding system [10–12]. In addition to *Corynebacterium pseudotuberculosis*, other pathogens such as, *Corynebacterium ulcerans*, Streptococcus spp., Staphylococcus spp. and *Corynebacterium pyogenes* were also reported to be involved in the development of CLA of camels [2,3,10,13,14].

The CLA in small ruminants and camelids is characterized by the formation of abscesses in one or more of the superficial lymph nodes and these may also extend to internal organs such as the lung and liver [1,4,15,16].

The CLA in camels was also reported to induce proliferation and congestion of lymph nodes without the development of abscesses [4,17]. However, moderate to severe abscessation of joints, subcutaneous, tissue, muscles and lungs were also reported. This differs from the findings in sheep where the abscessed were encapsulated with a thick capsule, containing odourless, non-granular, noncalcified, and thin creamy yellowish-white pus [17]. The pathognomonic onion ring appearance observed in sheep and goats were also detected in camels [2]. In addition, three different types of pyogranulomatous lesions have been observed in camelids, as a single abscess, a large central abscess with multiple small abscesses in the peripheral connective tissue or multiple abscesses [4].

Histologically, suppuration, necrosis, hyperplastic lymphoid follicles, and sinus histiocytosis of the affected lymph nodes were observed. The affected lung tissue showed suppurative foci with central necrosis and areas of acute inflammatory infiltrate around bronchioles beside congested vessels [18].

Diagnosis of caseous lymphadenitis is primarily based on a mixture of techniques including clinical observations of various external lymph nodes having abscesses, postmortem examination and bacterial culturing for isolation and identification of the pathogen. Biochemical reactions for the identification of the *Corynebacterium pseudotuberculosis* isolates vary in their fermenting ability as all strains produce acid but no gas from many carbon sources including glucose, fructose, maltose, mannose and, sucrose. Moreover, the bacterium was found to be negative for oxidase and positive for catalase and, urease [19,20]. However, most often biochemical confirmation is not conclusive due to the large phenotypic variability of *Corynebacterium pseudotuberculosis* [21]. Thus, PCR assay and 16S rRNA gene [22–24] sequencing are useful and reliable tools to confirm *Corynebacterium pseudotuberculosis* infection in animals [8,25].

It is known that, CLA is present in Old World Camelids (*Camelus dromedarius* & *Camelus bactrianus*) and New World Camelids (Llamas and alpacas) [26]. Moreover, the disease is also reported in Middle Eastern countries such as Iran [27], Saudi Arabia [28], Bahrain [29] and Jordan [17]. The aim of this study is to report and describe pathological findings associated with CLA in dromedary camels in the United Arab Emirates (UAE) and further to confirm the identity of the pathogen by using bacteriological and molecular techniques.

## Materials and methods

### Ethical approval

This research was approved by the research ethics committee Abu Dhabi Agriculture and Food Safety Authority (ADAFSA) (approval number: ADAFSA-EA-05-2021), and the study was done following the guidelines stated for animal use. A written consent (which was included in the sample request form approved by the ADAFSA research ethics committee) for the use of samples and animals was obtained from the camel’s owner before inclusion in the study.

### Clinical and necropsy sampling

A total of 12 adult male and female dromedary camels over five years of age from the three regions of the Abu Dhabi Emirate ((5 = Abu Dhabi, AD), 3 = Al Ain, AA) and 4 = Al Dhafra (Western Region, WR)) (S1 Fig) were examined by ADAFSA veterinarians and pathologists during routine clinical and postmortem examinations with special attention to the skin for all suspected animals of CLA disease. This study is based on cases examined between the periods from January 2015 to August 2020.

A total of 8 samples of blood and pus from clinical cases (n = 8 camels) and 8 samples of liver, lymph nodes, pus, muscle and lung at postmortem examination (n = 4 camels) were aseptically collected for laboratory analysis. Tissue samples were immediately fixed in 10% buffered formalin (Thermo Fisher Scientific, UK) according to [30] for histopathology while a copy of all samples including tissue sections were transported on ice to the ADAFSA veterinary laboratories for bacterial isolation and identification and further confirmation by 16S rRNA gene sequencing.

### Histopathological technique

Prescapular lymph node and liver tissues sections fixed in buffered formalin were processed in an automatic tissue processor (ATP1-220, USA) and embedded in paraffin blocks and cut into 5 μm thick sections. Sections were then stained with Haematoxylin and Eosin (H&E) (Thermo Fisher Scientific, UK) according to previous methods [30], and the images were acquired with Olympus digital microscope- U-APT (Olympus, Tokyo, Japan) to examine the histopathological changes.

### Bacterial isolation and identification

Samples transported on ice were cultured in readymade media: Blood agar, Nutrient agar as a general media and MacConkey agar (MAC), Columbia Naladixic Acid Agar (CNA), Mannitol salt agar (MSA) as a selective media (Pharmatrade, Dubai, UAE). According to previously described methods, the bacterial culture was incubated aerobically for 24 and 48 hours at 37°C [31]. The isolates were screened microscopically using Gram stain for the characteristic arrangements in a “V” formation (forming “Chinese letters”) as single or in pairs. To confirm a pure bacterial culture used for subsequent analysis, the plate was checked to contain only one single type of colony (yellowish white, opaque, hemolytic, and convex colonies). A Gram stain was prepared from the isolate and used to confirm the bacteria morphology, size and purity of the growth. The isolates were also studied biochemically with API Coryne (BioMérieux, Marcy l’Etoile, France) as per kit instruction, catalase test and nitrate reduction test and the isolates were further confirmed by using VITEK-MS (MALDI-ToF) system (BioMérieux, Marcy l’Etoile, France)

### Antimicrobial susceptibility testing

Antibiotic sensitivity testing (AST) using the disc diffusion method on Muller–Hinton Agar (Pharmatrade, Dubai, UAE) was performed to examine the sensitivity reactions of the isolates against Enrofloxacin (5 μg), Tetracycline (30 μg), Ampicillin (10 μg), Penicillin (10U) and Cotrimoxazole (Trimethoprim + Sulfamethoxazole 1.25/23.75 μg) (MAST Group Ltd, UK). *Staphylococcus aureus* ATCC 25923 was used as a positive control according to the Clinical and Laboratory Standards Institute [32]. The susceptibility of the isolates was determined according to the size of the inhibition zone.

### 16S rRNA gene sequencing

#### DNA extraction and amplification

DNA extraction was done from a pure culture of the isolate using the EZ1 DNA Tissue kit (Qiagen, Hilden, Germany) as per the kit instructions. Briefly, 4–6 colonies were harvested and lysed in 300 μL of buffer G2 at 56°C for 15 min. About 200 μL of the lysate was transferred to the Advanced EZ1 instrument and the DNA was eluted at 50 μL. DNA quality was measured on a Nanodrop 2000 spectrophotometer (Thermo Scientific, USA). PCR was performed with AmpliTag Gold 360 (Applied Biosystems, USA) to amplify part of the bacterial 16S rRNA gene (~ 700–900 bp) using the universal primer sets 27F (5ʹ-AGAGTTTGATCCTGGCTCAG-3ʹ) and 907R (5ʹ-CCCCGTCAATTCATTTGAGTTT-3ʹ) [33,34]. The master mix composed of 9.5 μL nuclease-free water,12.5 μL of AmpliTaq Gold Master Mix,1 μL of each forward and reverse primers (10pmol/μL). The template was added at volume of 1 μL to complete the total volume of the PCR mix to 25 μL. The PCR thermal profile was set to 95°C for 10 min for initial denaturation and activation of the polymerase followed by 40 cycles of 94°C for 30 s, 54°C for 30 s, and 72°C for 1 min. The final extension was done at 72°C for 7 min. The amplicons were visualized on a 1.5% agarose gel.

#### Sanger sequencing

The PCR products were purified with a QIAquick PCR Purification Kit (Qiagen, Hilden, Germany) as instructed. Bidirectional Sanger sequencing of the purified amplicons was performed with BigDye Terminator v3.1 Cycle Sequencing kit (Applied Biosystems) following the manufacturer’s guidelines for 20 μl reactions (5 ul of DNA, 8 ul of BigDye Terminator V3.1, 6 μL of water and 1 μL of primer) with an initial denaturation step at 96°C for 1 min, followed by 25 cycles of denaturation at 96°C for 10 s, annealing at 50°C for 5 s and extension at 60°C for 4 min. The reaction mixtures were purified with DyeEx 2.0 Spin Kit (Qiagen, Hilden, Germany). Sequencing was done on a SeqStudio Genetic Analyzer (Applied Biosystems), equipped with a cartridge containing a 28 cm column (Applied Biosystems), using the ‘LongSeq’ run module.

#### Assembly and mapping to the reference genome

The sequence trimming and assembly was performed with CLC Genomic Workbench v.20 (Qiagen, Aarhus, Denmark), and the consensus sequence (~700–900 bp) was subjected to a BLAST search of the NCBI RefSeq genome database (http://www.ncbi.nlm.nih.gov). Visualization of the reads mapped against the closest matching genome *Corynebacterium pseudotuberculosis* (GenBank accession number: NZ_CP054555.1) was performed using Unipro UGENE [35].

#### Sequence alignment and phylogenetic tree construction

For comparative sequence analysis of the 16S rRNA gene, 45 sequences of *Corynebacterium pseudotuberculosis*, *Corynebacterium renale*, *Corynebacterium diphtheriae* and *Corynebacterium ulcerans* were used. These sequences were found in previously published papers [36,37] or in nucleotide sequence databases. Multiple alignment of the sequences was carried out using Muscle implemented in MEGAX software [38]. The tree was built with Maximum Likelihood method and Kimura 2-parameter model [39] with 1000 Bootstrap confidence using MEGAX software [38]

## Results

### Clinical history

Clinically, camels showed enlargement of the inferior cervical (cervicales laterals) and prefemoral lymph nodes (iliaci externi). In addition, chronic weight loss, reduced appetite, emaciation, no response to antibiotics treatment, recumbence and death were also observed. From the 12 camels examined, four camel carcasses (2 from Abu Dhabi (AD), and two from Al Ain (AA)) were necropsied and revealed emaciation and presence of multiple external and internal abscesses, particularly in the lungs and the liver.

### Post-mortem examination results

**Fig 1:**
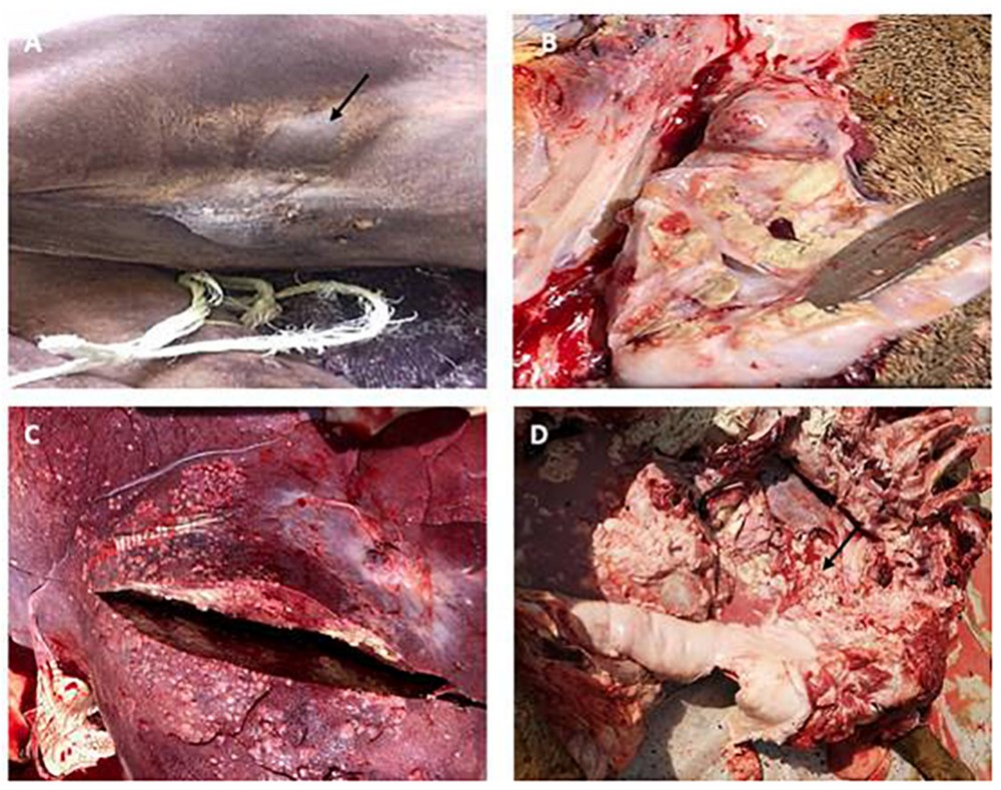
Gross lesions of CLA in dromedary camel. (A) superficial abscesses on the right prefemoral lymph nodes (iliaci externi) (arrow). (B) Incised right inferior cervical lymph node (cervicales laterals) showing multiple caseated caseous pyogranulomas containing yellowish-cheesy martials. (C) Liver showing multifocal-diffused pyogranulomas contained yellowish- cheesy pus. (D) Thoracic cavity showed multiple open large abscesses in the lung with adhesion to the chest wall (arrow).

Gross lesions observed at postmortem examinations showed severe emaciation and absence of internal body fat. The inferior cervical (cervicales laterals) lymph nodes were enlarged with multiple tubercles of variable size having caseous material; abscesses were also seen in subcutaneous tissues in the lateral and medial aspects of the proximal part of the hindquarters and biceps femoris muscles. Multiple large similar caseous abscesses were also found in internal organs, particularly in the lung and liver. The abscesses were encapsulated by a relatively thick layer of necrotic and fibrous tissues. They contained odorless, non-granular and non-calcified thin creamy yellowish to white pus (Fig 1).

### Histopathological findings

**Fig 2:**
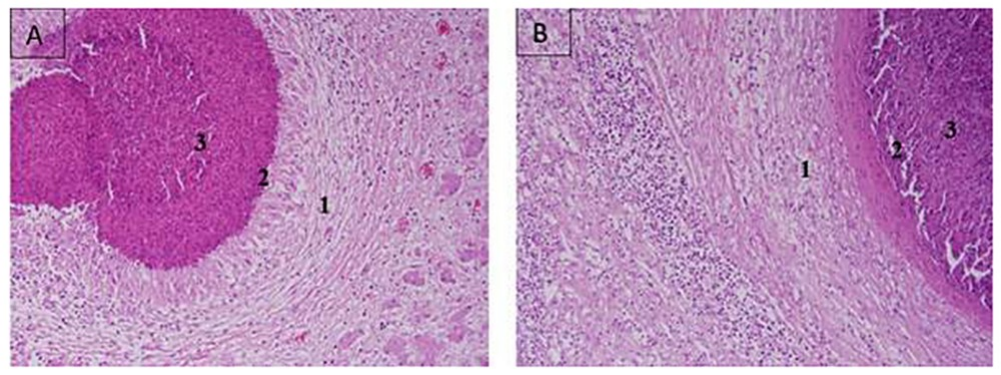
Histologic section of liver and lymph node of dromedary camel infected with CLA. (A) Liver section showing central caseous necrotic core admixed bacterial colonies and infiltration of polymorphonuclear cells, lymphocytes, plasma cells and macrophages (3), surrounded by pyogenic membrane (2), and fibrous capsule (1), H&E stain, 20×. (B) Lymph node section showing central caseous necrotic abscess (3), surrounded by inflammatory cells (2) and bound by a thick fibrous capsule (1), H&E stain 20 ×.

Microscopic lesions of the pyogranulomatous *Corynebacterium pseudotuberculosis* abscesses are composed of a central necrotic core of pus. Infiltration of chronic inflammatory cells (lymphocytes, plasma cells and macrophages) surrounded by a pyogenic membrane, and outer zone of fibrous connective tissue capsule is shown in liver and lymph node sections (Fig 2).

### Bacterial culture results

*Corynebacterium*.*pseudotuberculosis* was isolated from all samples collected (n = 12). Pure yellowish-white, opaque, α- hemolytic and convex colonies were seen on Blood agar and Columbia Naladixic Acid Agar (CNA). Small Gram positive cocco-bacilli arranged in Chinese letters were seen under the microscope using Gram stain. Isolates were confirmed as *Corynebacterium pseudotuberculosis* with VITEK-MS (MALDI-ToF) (BioMérieux) with a confidence level of 99.9% (S2 Fig). All isolates tested with API bionumber profile (0111324 & 0101324) were catalase-positive and nitrate negative. Nitrate reduction yielded a negative result for all the isolates, which concluded that the isolates were type I strain (biovar ovis).

### Antimicrobial susceptibility results

Antibiotic sensitivity testing showed that all the isolates were sensitive to Enrofloxacin (5 μg), Tetracycline (30 μg), Ampicillin (10 μg), Penicillin (10U) and Co-trimoxazole (Trimethoprim + Sulfamethoxazole 1.25/23.75 μg) at the indicated concentrations.

### Molecular results

#### The 16S rRNA gene sequence analysis

**Fig 3:**
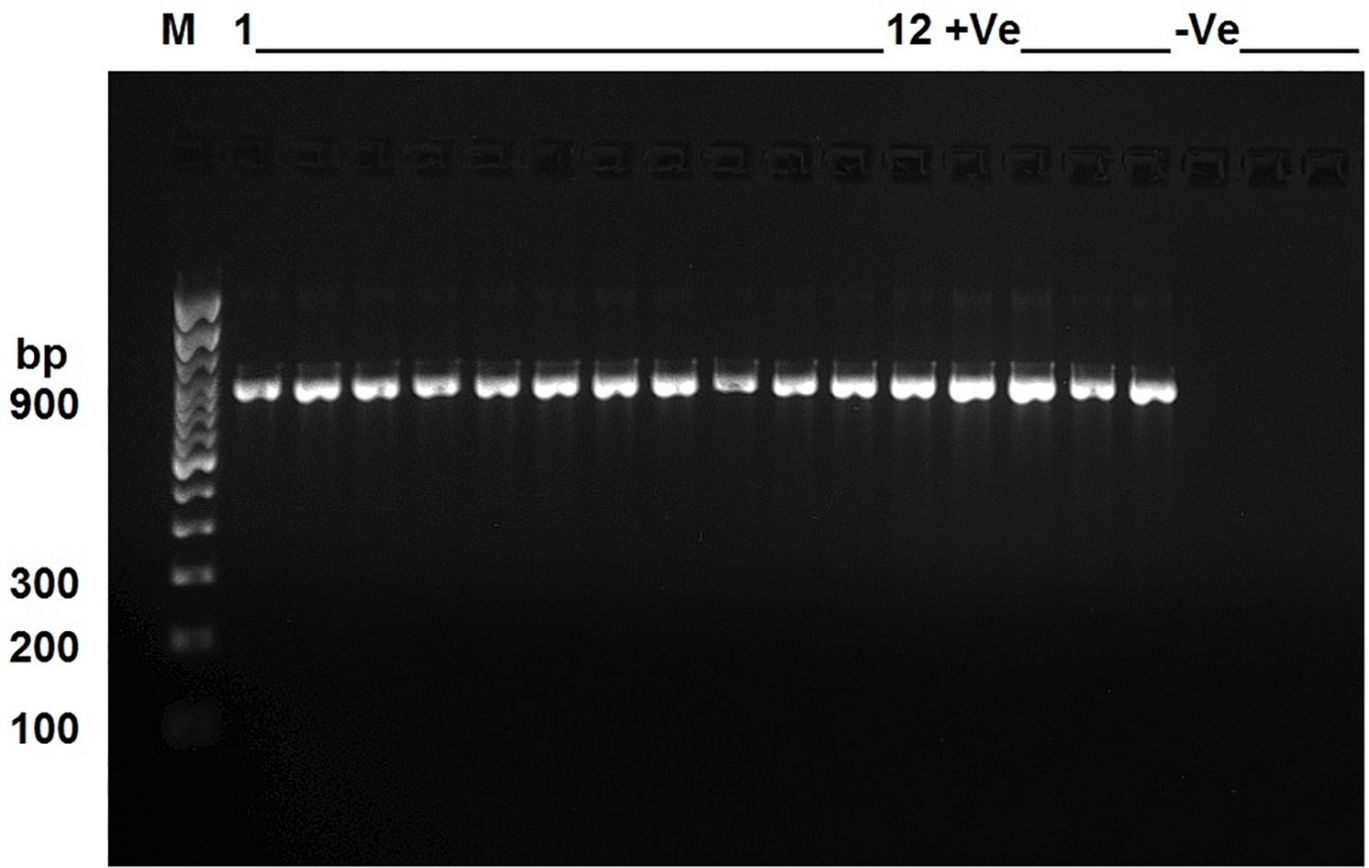
Gel image of PCR amplified 16S rRNA gene (partial, ~ 900 bp) for the 12 samples shown in Table 1.

**Fig 4:**
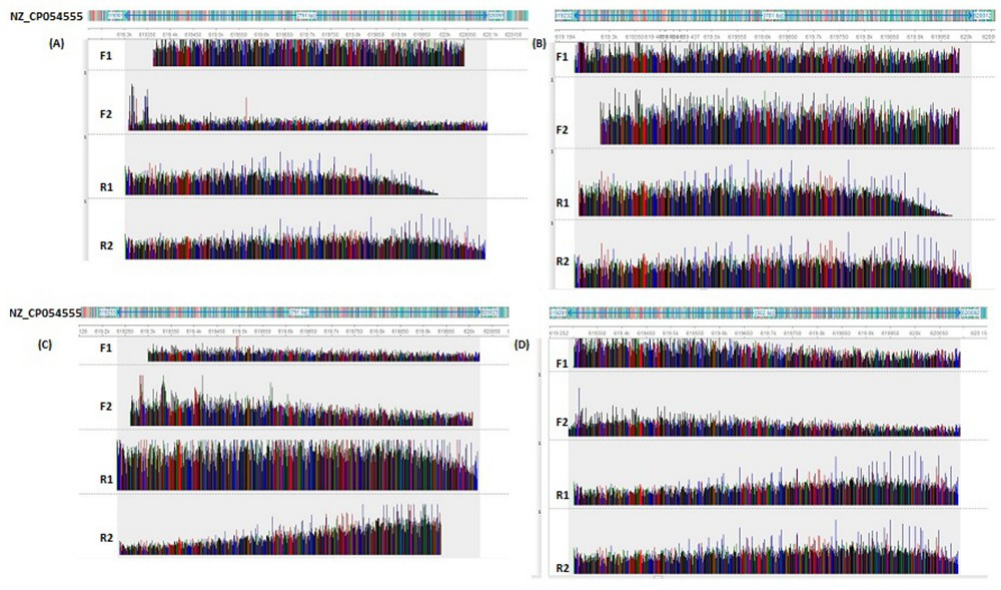
Sequence mapping. Images of mapping of sequence reads for 4 representative samples (sample 1, 7, 9 and 12) to the reference genome of *Corynebacterium pseudotuberculosis* (Accession number: NZ_CP054555.1). The sequence reads mapped to reference genome between positions 619301–620091 (791 bp), 619232–620012 (781 bp), 619233–620023 (791 bp) and 619293–620093 (801 bp) for A (sample 1), B (sample 7), C (sample 9) and D (sample 12) respectively.

The gel image of the partial amplification of the 16S rRNA gene was shown in (Fig 3 and S1 Raw images). Blast search of the 12 16S rRNA gene sequence (~700–900 bp) obtained from the isolates, revealed a 100% identity to *Corynebacterium pseudotuberculosis* sequence available in the NCBI database. The top three close sequences to our isolates retrieved from Blast analysis were shown in Table 1. Mapping of the isolates reads to the reference sequence of *Corynebacterium pseudotuberculosis* (Accession number: NZ_CP054555.1) indicated a 100% % similarity to the reference genome. Information on the length of the 16S rRNA gene sequences per isolate along with the regions covered in the reference genome was shown in Table 1. We have further shown a mapping chromatogram for four representatives isolates (1, 7, 9 and 12) in (Fig 4). These *Corynebacterium pseudotuberculosis* isolates were given strain names (CRY1-CRY12) and their 16S rRNA gene partial nucleotide sequences were deposited in the GenBank under the accession numbers shown in (Table 2).

**Table 1:** The top three close sequences of the *Corynebacterium pseudotuberculosis* to our isolates, retrieved from BLAST analysis.

S/N	Strain	GenBank	Host	Isolation source	Country	Collection date
1	AHDP:2020	MT649220.1	*Camelus dromedarius*	Abscess	India	2020
2	PA09	CP054555.1	NA	Caseous material	Brazil:Para	2018
3	WM M 5	LC549531.1	*Capra aegagrus hircus*	NA	Malaysia	2013

NA = not available.

**Table 2:** GenBank accession numbers for the 16S rRNA gene partial sequence of *Corynebacterium pseudotuberculosis* isolated from dromedary camels in UAE (2015–2020) along with the length of each isolate sequence and the span on the reference genome *Corynebacterium pseudotuberculosis* (Accession number: NZ_CP054555.1) during mapping.

S/N	GenBank submission	Strain	Accession number	Length(bp)	Span
1	SUB8562986	CRY1	MW276079	791	619301–620091
2	SUB8562986	CRY2	MW276080	746	619247–619993
3	SUB8562986	CRY3	MW276081	750	619234–609983
4	SUB8562986	CRY4	MW276082	758	619287–620044
5	SUB8562986	CRY5	MW276083	784	619232–620015
6	SUB8562986	CRY6	MW276084	805	619236–620040
7	SUB8562986	CRY7	MW276085	781	619232–620012
8	SUB8562986	CRY8	MW276086	778	619237–620014
9	SUB8562986	CRY9	MW276087	791	619233–620023
10	SUB8562986	CRY10	MW276088	770	619267–620035
11	SUB8562986	CRY11	MW276089	746	619244–609988
12	SUB8562986	CRY12	MW2760790	801	619293–620093

The chromatogram mapping of the isolates in bold (S/N 1, 7, 9 and 12) was shown in (Fig 4).

#### Phylogenetic analysis of *Corynebacterium pseudotuberculosis*

**Fig 5:**
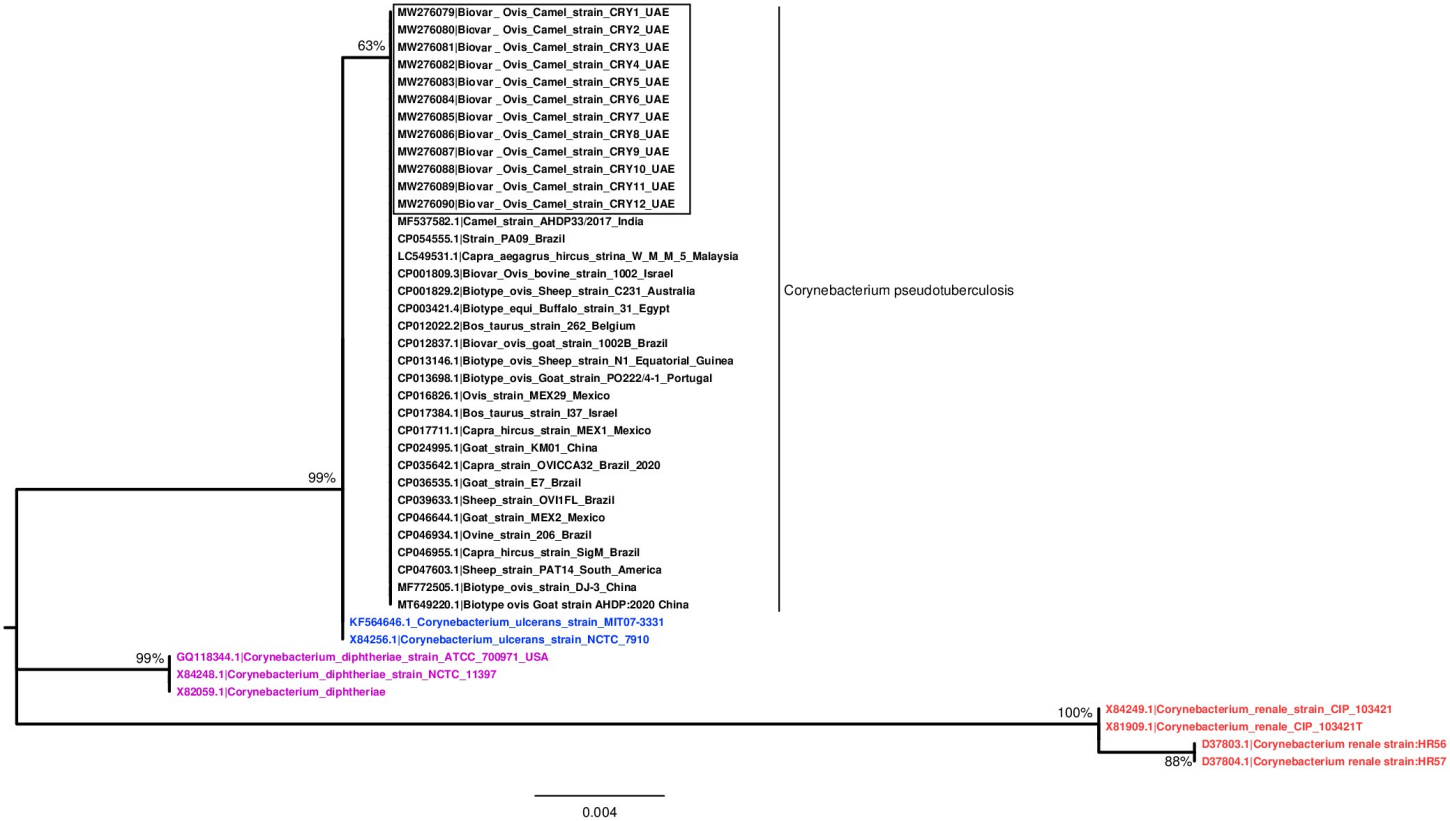
Phylogenetic tree by Maximum Likelihood method. The tree based on the sequence of 16S rRNA gene was constructed by MEGAX software [38] using Maximum Likelihood method based on the Kimura 2-parameter model [39]. We used a Boostrap value of 1000 repetition, *Corynebacterium pseudotuberculosis* isolates (Black Square bordered) were included in this study as well as GenBank reference samples of the subspecies ovis and equi, a reference sequence of the *Corynebacterium ulcerans* (Blue), *Corynebacterium renale* (Red) and *Corynebacterium diphtheria* (Pink).

Thirty-three sequences of 16S rRNA gene obtained from other publications (described in methodology) or GenBank along with 12 partial sequences obtained from *Corynebacterium pseudotuberculosis* isolates in this study (in total 45 sequences) were used in the phylogenetic analysis (GenBank accession numbers of the sequences used were included in the tree). Phylogenetic analysis of the 16S rRNA gene can easily separate *Corynebacterium pseudotuberculosis* from other *Corynebacterium* spp., with less power to differentiate biovar ovis from biovar equi (Fig 5), consistent with previous studies [36,37]. *Corynebacterium ulcerans* was found to be the closest species to the *Corynebacterium pseudotuberculosis* as observed earlier [36,37].

## Discussion

The present study describes the pathological, bacteriological and molecular analysis of a caseous lymphadenitis (CLA) in dromedary camels in three regions of Emirate of Abu Dhabi, UAE (Abu Dhabi (AD), Al Ain (AA) and Al Dhafra (Western Region, (WR)). The affected camels were either in the farm or brought to the ADAFSA veterinary laboratory for a routine postmortem examination.

The external form seen is the most frequent type of CLA in small ruminants, which is characterized by abscess formation in superficial lymph nodes and subcutaneous tissues [40]. Similar findings of external CLA in camels were also reported and this form was found to be more prevalent than the visceral form [16]. However, in our study, both external and internal lesions of CLA were observed which could be explained by the method of transmission of the disease, as CLA infection spread via ingestion, inhalation or through wounds [17].

In sheep, the lymph nodes with characteristics pathognomonic lesions (onion ring shape) of *Corynebacterium pseudotuberculosis* infection is common [41] and similar result have also been reported in camels liver [2].

The lesions of necropsied dromedary camels observed in the liver, lungs, and lymph nodes were found to be of different sizes: miliary tubercles in the liver, a large a central abscesses of creamy white cores with multiple small abscesses in the peripheral connective tissue capsule in the lungs and the lymph nodes with creamy white cores. Such postmortem changes due to *Corynebacterium pseudotuberculosis* infection were also reported in dromedary camels in Jordan [17]. However, various sized abscesses of *Corynebacterium pseudotuberculosis* randomly scattered in the liver lobes characterized by thick, whitish, caseous, and lamellar material were the only lesions reported in one slaughtered camel in Iran [42].

Histopathological examination of tissue sections obtained from visceral organs (lymph nodes and liver) revealed extensive abscess formation (central layers of caseous necrosis and calcification surrounded by a fibrinous wall), due to *Corynebacterium pseudotuberculosis* infection in dromedary camels [42] as well as in small ruminants [41]. Similar microscopic findings are also found in lymph node and liver sections stained with H&E in the present study.

In the present study, *Corynebacterium pseudotuberculosis* isolated from all external and internal visceral organs is associated with caseous necrosis in these organs. These results are in line with previous reports in camels and in small ruminants [28]. However, in one study, although *Corynebacterium pseudotuberculosis* was isolated from superficial and internal visceral lymph nodes, there was no detectable caseous necrosis or lamination found [17].

Bacterial culture successfully isolated *Corynebacterium pseudotuberculosis* from all samples tested. The bacterial isolate from a liver of a dromedary camel with multiple pyogranulomatous lesions is similar to previous findings that isolated the organism from liver abscesses in mature alpaca and dromedary camels [43,44].

The isolates of *Corynebacterium pseudotuberculosis* were shown to be sensitive to all antibiotics tested, and this observation may indicate that the pathogen was not exposed to any antibiotics and therefore did not experience any selection pressure that may have caused mutations. Although *Corynebacterium pseudotuberculosis* isolates were sensitive to all antibiotics used for *in vitro* antibiotic susceptibility tests, CLA may continue to be a serious problem for animal breeders because treatment with these antibiotics is generally not effective *in vivo* due to several factors, including the protective nature of the bacterial capsule, the formation of the encapsulated abscess as well as the intracellular existence of the microorganism [17,45–47]. Therefore, prophylactic and therapeutic treatment won’t ensure that animals are free from infection with this microorganism. Hence, these infected animals may serve as a reservoir of infection. As a result of this, the most practical method of controlling CLA in small ruminants is to cull all animals with the palpable lesions [48]. However, this is impracticable to dromedary camels because of their economic and cultural value. In such a situation, it is recommended to perform both surgical and antibiotic treatment.

Vaccines against CLA for sheep and goats are commercially available. However, these vaccines are not commonly used in dromedaries because the protective doses are not adopted for camels as they are not evaluated for use in camels. They likewise do not provide complete protection against the formation of abscess. Besides, these vaccines produced granulomas during vaccination trials [4]. A new type of vaccine composed of a mixture of 2 CLA biotypes (ovine/caprine and equine/bovine) has given complete protection in dromedary camels in vaccination trails, however, more research is needed to prove its use commercially [4]. *Corynebacterium pseudotuberculosis* type 1 or biovar ovis was isolated from camels, Hawari [17] suggested that infection was transmitted to camels from infected sheep and goats grazing together on the same pasture [17].

Both biotypes of *Corynebacterium pseudotuberculosis*: ovine/caprine (nitrate negative) and equine/bovine (nitrate positive) have been reported in dromedaries using the nitrate reduction test [10,49]. However, in the present study, only biotype ovis is identified by the nitrate reduction test which yielded a negative result for all the isolates. This result was further supported by BLAST analysis of the 16S rRNA gene partial sequences obtained from our isolates, which showed that the *Corynebacterium pseudotuberculosis* (Accession number: LC549531.1) isolated from *Capra aegagrus hircus* is one of the three identical sequences close to our isolates (Fig 5 and Table 1). This would indicate that preventive measures should be taken into consideration when dromedary camels are in contact with sheep and goats, horses or cattle affected by *Corynebacterium pseudotuberculosis* bio-type ovis or equi in all cases [49]. To avoid any infection from small ruminants, camels should not be intermingled with sheep and goats, horses or cattle. It is obvious from phylogenetic analysis, the 16S rRNA gene sequence could only be useful to separate *Corynebacterium pseudotuberculosis* from other *Corynebacterium* spp., but not to differentiate biovar ovis from biovar equi [36,37]. This differentiation step could further be done using *fusA* [36] or *rpoB* gene [50]. The close phenotypic and genotypic relationship among *Corynebacterium pseudotuberculosis* isolates from various countries in the world and among different animal species, might be evidence that the infections had a common source [51].

### Conclusions

In conclusion, the results of the present investigation indicate that caseous lymphadenitis is prevalent as sporadic cases among dromedary camels in the Emirate of Abu Dhabi, (12 cases by routine diagnosis). As the isolates were shown to be sensitive to all antibiotics tested *in vitro*, and practically the infected camels did not respond to antibiotics, this may encourage combining other preventive and control measures such as good sanitary and hygiene in addition to vaccination to control *Corynebacterium pseudotuberculosis* infection in camels as well as small ruminants.

## Supporting information

S1 Fig Map of the United Arab Emirates (UAE). The location of three regions of the study: Abu Dhabi region, AD (orange color), Al Dhafra region or Western region, WR (Pink color) and Al Ain, AA (green color) in the Emirate of Abu Dhabi (black line bordered) was shown. Image obtained from: https://www.shutterstock.com.
(TIF)

S2 Fig VITEK-MS (MALDI-ToF). (BioMérieux, Marcy l’Etoile, France), confirmation of *Corynebacterium pseudotuberculosis* isolates with a confidence value of 99.9%.
(TIF)

S1 Raw images A raw gel image of Fig 3 showing PCR amplified 16S rRNA gene (partial, ~ 900 bp) for the 12 samples used in the study. (TIF)
