# Supplementary figures and images for: Pathology, bacteriology and molecular studies on caseous lymphadenitis in Camelus dromedarius in the Emirate of Abu Dhabi, UAE, 2015-2020
Source: PLoS One. 2021 Jun 8;16(6):e0252893. doi: 10.1371/journal.pone.0252893 (PMC8186769; doi:10.1371/journal.pone.0252893)

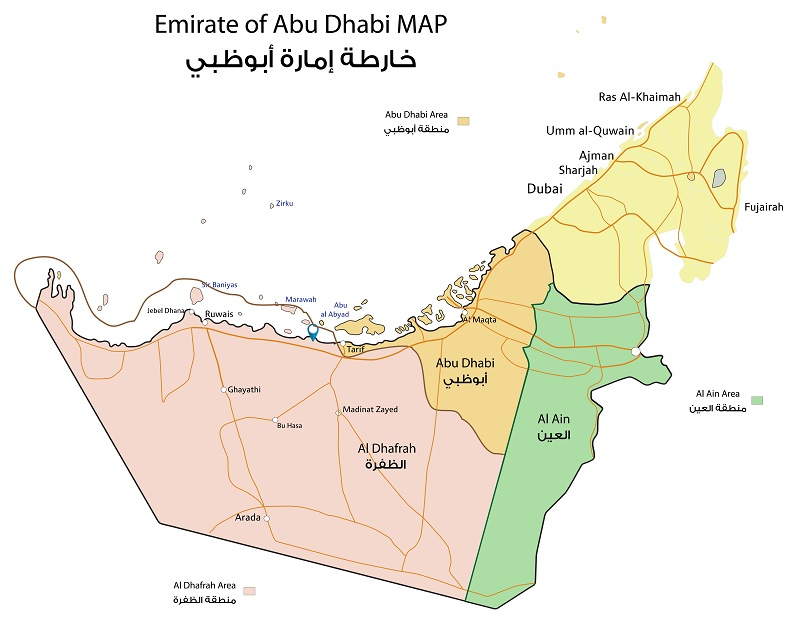

Supplement: S1 Fig — The location of three regions of the study: Abu Dhabi region, AD (orange color), Al Dhafra region or Western region, WR (Pink color) and Al Ain, AA (green color) in the Emirate of Abu Dhabi (black line bordered) was shown. Image obtained from: https://www.shutterstock.com. (TIF) [file pone.0252893.s001.tif]

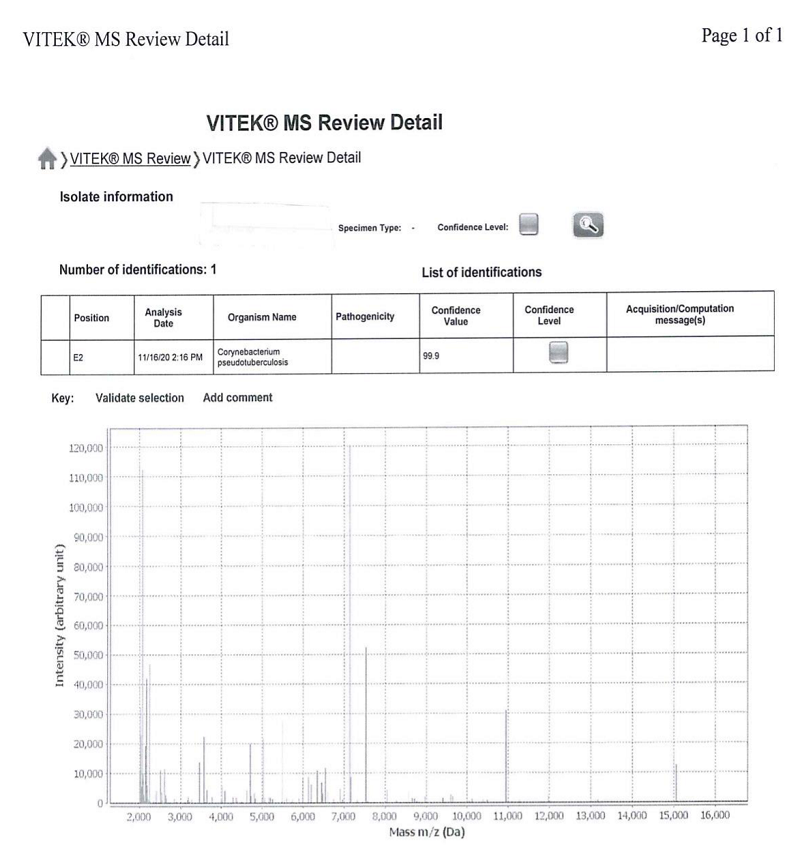

Supplement: S2 Fig — (BioMérieux, Marcy l’Etoile, France), confirmation of Corynebacterium pseudotuberculosis isolates with a confidence value of 99.9%. (TIF) [file pone.0252893.s002.tif]

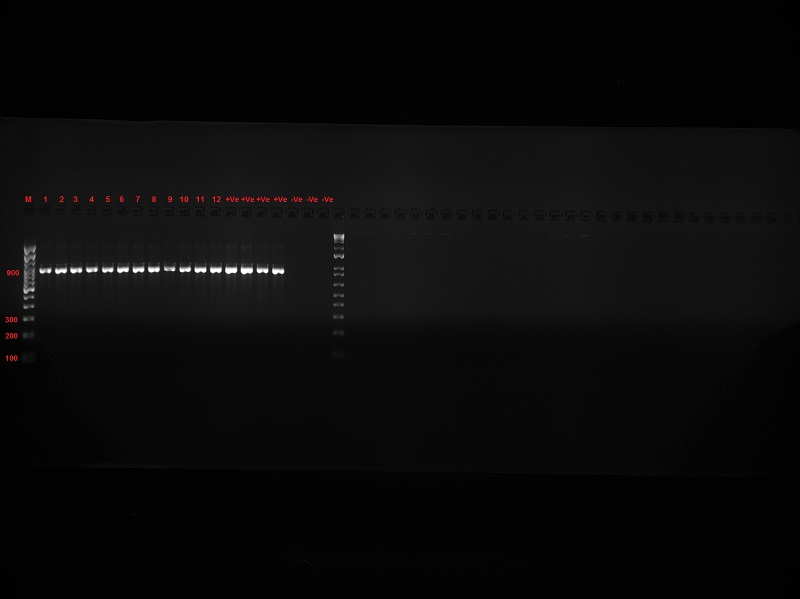

Supplement: S1 Raw images — (TIF) [file pone.0252893.s003.tif]
